# Whole genome sequencing revealed host adaptation-focused genomic plasticity of pathogenic *Leptospira*

**DOI:** 10.1038/srep20020

**Published:** 2016-02-02

**Authors:** Yinghua Xu, Yongzhang Zhu, Yuezhu Wang, Yung-Fu Chang, Ying Zhang, Xiugao Jiang, Xuran Zhuang, Yongqiang Zhu, Jinlong Zhang, Lingbing Zeng, Minjun Yang, Shijun Li, Shengyue Wang, Qiang Ye, Xiaofang Xin, Guoping Zhao, Huajun Zheng, Xiaokui Guo, Junzhi Wang

**Affiliations:** 1 Key Laboratory of the Ministry of Health for Research on Quality and Standardization of Biotech Products, National Institutes of Food and Drug Control, Beijing 100050, People’s Republic of China; 2 Department of Microbiology and Immunology, Institute of Medical Science, Shanghai Jiao Tong University School of Medicine, 280 South Chongqing Road, Shanghai 200025, People’s Republic of China; 3 Laboratory of Medical Foods, Shanghai Institute of Planned Parenthood Research, 2140 Xie-Tu Road, Shanghai 200032, People’s Republic of China; 4 Shanghai-MOST Key Laboratory of Health and Disease Genomics, Chinese National Human Genome Center at Shanghai, Shanghai Zhang Jiang Hi-TechPark, 250 Bi-Bo Road, Shanghai 201203, People’s Republic of China; 5 Department of Population Medicine and Diagnostic Sciences, Cornell University, Ithaca, New York, 14853, United States of America; 6 Key Laboratory of Synthetic Biology, Institute of Plant Physiology and Ecology, Shanghai Institutes for Biological Sciences, Chinese Academy of Sciences, Shanghai 200032, People’s Republic of China; 7 State Key Laboratory for Infectious Disease Prevention and Control, National Institute for Communicable Disease Control and Prevention, Chinese Centre for Disease Control and Prevention, 155 Changbai Road, Changping District, 102206 Beijing, People’s Republic of China; 8 Guizhou Provincial Centre for Disease Control and Prevention, 73 Bageyan Road, Guiyang 550004, People’s Republic of China

## Abstract

Leptospirosis, caused by pathogenic *Leptospira* spp., has recently been recognized as an emerging infectious disease worldwide. Despite its severity and global importance, knowledge about the molecular pathogenesis and virulence evolution of *Leptospira* spp. remains limited. Here we sequenced and analyzed 102 isolates representing global sources. A high genomic variability were observed among different *Leptospira* species, which was attributed to massive gene gain and loss events allowing for adaptation to specific niche conditions and changing host environments. Horizontal gene transfer and gene duplication allowed the stepwise acquisition of virulence factors in pathogenic *Leptospira* evolved from a recent common ancestor. More importantly, the abundant expansion of specific virulence-related protein families, such as metalloproteases-associated paralogs, were exclusively identified in pathogenic species, reflecting the importance of these protein families in the pathogenesis of leptospirosi*s*. Our observations also indicated that positive selection played a crucial role on this bacteria adaptation to hosts. These novel findings may lead to greater understanding of the global diversity and virulence evolution of *Leptospira* spp.

Leptospirosis, caused by pathogenic spirochetes of the genus *Leptospira*, is one of the most widespread and significant zoonotic diseases in the world^1^. A wide variety of mammalian hosts can serve as infection reservoirs. Human disease is usually acquired following environmental exposure to *Leptospira* shed in the urine of infected animals, or through occupational exposure to contaminated soil and water^1^. The estimated number of human leptospirosis cases averages over 500,000 per year, and the mortality rate can reach up to 25%^1^,^2^. Moreover, the incidence is expected to increase further with anticipated global warming. Therefore, leptospirosis has the potential to become even more prevalent and has recently been recognized as an emerging infectious disease^1^.

Currently, twenty *Leptospira* species, comprised of pathogenic, intermediate and saprophytic groups, have been identified based on classical DNA-DNA hybridization studies and 16S ribosomal RNA gene phylogeny^3^,^4^. The pathogenic group consists of nine species, *i.e., Leptospira interrogans, L. borgpetersenii, L.kirschneri, L. alexanderi, L. alstonii, L. kmetyi, L. noguchii, L. santarosai*, and *L. weilii*^3^. Until now, five intermediate *Leptospira* species have been reported, which occasionally cause disease in humans and animals^3^,^5^,^6^. Six saprophytic species, such as *L. biflexa*, are not pathogenic^7^. Recently, genome sequencing of several *Leptospira* species has revealed high-level genomic plasticity of the genus^7^,^8^,^9^,^10^,^11^,^12^,^13^. *L. interrogans* and *L. borgpetersenii* genomes contain approximately 3,400 and 2,800 predicted coding genes, respectively, of which 656 are pathogen-specific and not found in the saprophyte *L. biflexa*^7^,^10^,^11^. The functions of most (59%) of these genes are unknown, reflecting the presence of pathogenic mechanisms unique to *Leptospira* spp. Comparative genomic analysis also suggests that pathogenic genospecies of *Leptospira* have a common progenitor with a genome resembling that of *L. biflexa*^7^. Furthermore, the genome reduction in *L. borgpetersenii* also reflects the increase of host dependence for surviving in different host-determined environmental conditions^11^.

The apparent correlation between the biodiversity of *Leptospira* spp and their hosts infers that host adaptation might be the driving force of *Leptospira* diversification and evolution^3^,^11^. However, this hypothesis has not been supported by appropriate studies designed to dissect the evolutionary mechanisms in *Leptospira*. Therefore, to improve our understanding of genetic diversity at the whole-genome level for *Leptospira* species that reside in distinct habitats, and to gain insights into their evolutionary path, here we detail comparative genomic and phylogenomic analyses of 102 newly sequenced pathogenic *Leptospira* genomes from isolates found throughout the world with a previous published strain.

## Results and Discussion

### General genomic information suggested high diversity among *Leptospira* species

We sequenced 102 genomes of pathogenic *Leptospira* isolated from 13 countries and districts from 6 continents, including eight known pathogenic species (Supplementary dataset S1). These strains contained 78 *Leptospira* serovars circulating throughout the world. A total of 72 isolates from Chinese domestic sources representing the full spectrum of genetic diversity were selected for this study. Of these isolates, 67 were from humans, while the remaining isolates were from various animal hosts, such as rodents, frogs, domesticated dogs, pigs, and cattle. Thus, it is assumed that these genomes provide a comprehensive representation of the geographic and genetic diversity of *Leptospira* for understanding the global diversity and evolution of this genus of spirochetes.

The sequence reads were assembled into genomes with an average coverage per genome of 353–fold. General information for all genomes is summarized in Supplementary dataset S2. Among the eight pathogenic *Leptospira* species sequenced, the largest genome was that of *L. interrogans*, at 4.77 Mb (4.40–5.02 Mb); this was approximately 900 Kb larger than the smallest genome (*L. borgpetersenii*; 3.86 Mb, range: 2.96–3.62 Mb). Interestingly, the range of the diversity of genome sizes in *Leptospira* spp. (1,231 kb) is much higher than that in other zoonotic pathogens, such as *Yesinia pestis* (305 kb, average genome size = 4.5 Mb)^14^ and *Brucella* species (293 kb, average genome size = 3.3 Mb)^15^. The G + C contents of the 102 genomes ranged from 34.9% to 42.5% (Table 1).

The similarity between any pair of species was determined by summation of all identities found in high-scoring segment pairs (HSPs) divided by the total genome length (Supplementary Figure 1). Our analysis showed that pair-wise genome sequence similarity varied from less than 1% to 79.3% across 18 *Leptospira* species, including the eight pathogenic species sequenced in this study, four saprophytic species, five intermediate species and *L. kmetyi*, a newly described pathogenic species (Supplementary dataset S3). Such high diversity suggests that distinct mechanisms may have been involved in the evolution of different *Leptospira* spp.

### The large pan-genome of *Leptospira*

**Figure 1:**
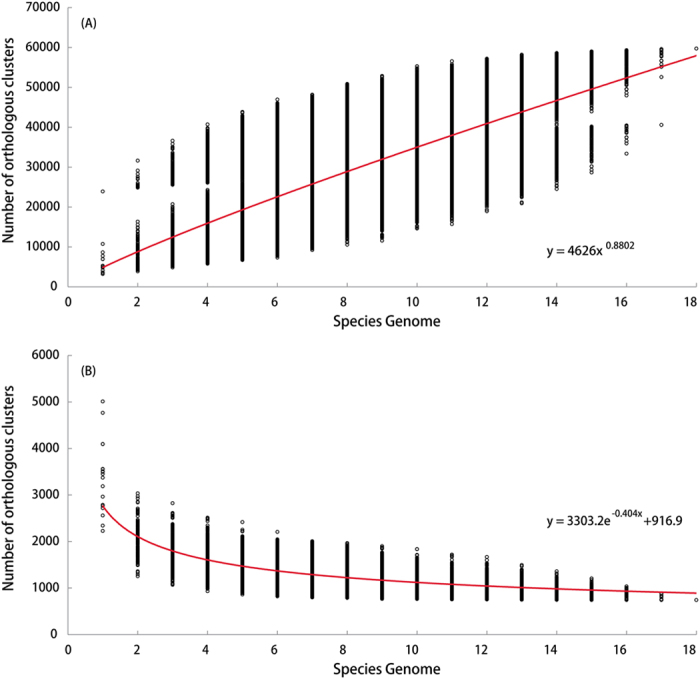
Accumulation curves for the pan-genome (**A**) and core-genome (**B**) of 18 *Leptospira* species. Circles represented number of ortholog clusters for the different strain combinations. The red curve of panel A was a least squares fit of the power law y = k x^γ^ to medians, with the exponent γ > 0 indicating an open pan-genome. The red curve of panel B was least squares fit of the exponential decay y = kc exp[−x/tc] + Ω to medians, with Ω representing extrapolated core genome size. The equation was illustrated in each panel.

The pan-genome curve of selected strains from 18 *Leptospira* species was created, which fit a power law function with an exponent γ = 0.88, and did not appear to reach saturation (Fig. 1A). According to the Heaps’ law model^16^, if the power law exponents γ > 0, the pan-genome is considered open^17^, which is typical of species colonizing multiple environments and having multiple ways of exchanging genetic material^18^, such as *Leptospira* species adapting to a wide variety of mammalian hosts and having large genome diversity. The predominant *Leptospira* species, *i.e., L. interrogans* and *L. borgpetersenii*, also displayed an open pan-genome (Supplementary Figure 2).

In contrast to the pan-genome (57,765 pan genes) of the 18 *Leptospira* species, the number of the core-genome was kept relatively constant (1,023 core genes) (Fig. 1). Further analysis identified 1,438 annotated core genes in all pathogenic *Leptospira* species (Supplementary dataset S4), accounting for only 0.3% of a total 47,100 in the pathogenic *Leptospira* pan genome. We identified a total of 780 virulence-related genes in the *Leptospira* pan-genome, and 287 of them were significantly enriched in core genes [false discovery rate (FDR) = 1.85E–235]. 20% of these core genes were related to encoding toxins and virulence factors. Meanwhile, we also re-constructed the pathogenic *Leptospira* pathway using the pan-genome, and found significant enrichment (all FDR < 0.05) of core genes in fundamental metabolic pathways (*e.g.*, pyruvate metabolism, carbon fixation, fructose and mannose metabolism and glutathione metabolism) and bacterial chemotaxis (Supplementary dataset S5). Motility and chemotaxis are required for pathogenic species in order to colonize and invade a host^19^.

### More genes were lost than gained before separation of pathogenic and intermediate groups

**Figure 2:**
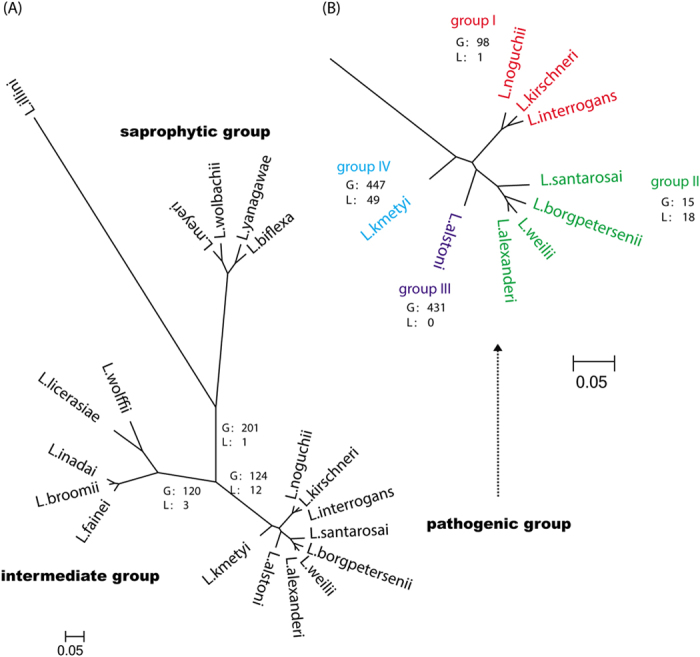
Phylogenetic analysis based on the maximum likelihood of the concatenated core genes of the *Leptospira* genome with *Leptonema illini* as the outgroup. (**A**) All spirochete species genomes were included. (**B**) The enlarged pathogenic group of from the phylogenetic tree. Numbers before and after slash showed the numbers of gains and losses, respectively (G: gain; L: loss). There were gene gain and loss events in the evolution from the root to the lineages. Scale bar indicated an evolutionary distance of 0.05 amino acid substitutions per position.

*L. biflexa* is a soil bacterium with a genome of 3.95 Mb that cannot replicate intracellularly and causes no infection; previous phylogenomic analyses speculated the common progenitor of intermediate and pathogenic *Leptospira* spp was a strain with a genome similar to that of *L. biflexa*^7^,^8^. To study the evolutionary process from *L. biflexa* to pathogenic *Leptospira*, we performed a phylogenetic analysis based on the core-genome of all *Leptospira* spp. using a closely related spirochete (*Leptonema illini*) as the outgroup (Fig. 2A and Supplementary Figure 3). The results showed that intermediate and pathogenic isolates of the family *Leptospiraceae* formed the two deepest branches while saprophytic species were located near the most recent common ancestor (MRCA), which indicated that virulent traits favoring host infection have been acquired independently during the evolution of the genus.

To avoid the gene gain/loss events of *L. biflexa* affecting our analysis, we used the pan-genome of saprophytic groups representing the MRCA to study the gene gain events in the pathogenic and intermediate groups, while the core-genome of saprophytic groups was used to study gene loss events. Further analysis showed that *Leptospira* spp. lost 383 MRCA genes and gained 281 genes before intermediate and pathogenic groups diverged (Fig. 2A). Among the lost genes, 46 were involved in metabolic pathways, and an enrichment in two-component system (TCS) (FDR = 0.003) was observed (Supplementary dataset S6). TCS comprised of the sensor kinase and response regulator play the important role for allowing bacteria to sense, respond, and adapt to changes in their environment or in their intracellular state^20^. The loss of two-component system indicated that distinct signal transduction systems existed between the saprophytic and pathogenic species. Such loss might be the first step in the evolution from strains capable of surviving in complex ambient environments into those adapted for pathogenic life. Indeed, genes that are no longer required in new environments are often lost when pathogens (such as *Yersinia pesti* and *Bordetella pertussis*) have adapted to an human ecological niche^21^,^22^, and the balance between gene gain and loss were common in pathogenic strain evolution. The phenomenon of net gene loss in the process of pathogenic *Leptospira* spp evolution was also observed in other pathogens like *B. pertussis*, which may have undergone gene loss occurring during the evolution path from a *Bordetella bronchiseptica*-like progenitor, continuing through to the emergence of *B. pertussis*^23^.

### Gene acquisition in pathogenic strain evolution contributing to host adaption

The core-genome of each pathogenic *Leptospira* species varied from 2,639 to 3,818, with 2,101 to 2,257 genes inherited from MRCA, occupying 46–50% of pathogenic *Leptospira* species genes (Supplementary dataset S7). The average gene number loss in the pathogenic group (1,382) was estimated to be larger than that in the intermediate group (1,241, ranging from 1,192 to 1,291), coinciding with the relatively long evolutionary distance of the pathogenic group. Although the lost MRCA genes varied in each species, pathway enrichment analysis indicated significant enrichment (all FDR < 0.001) of these lost genes in starch and sucrose metabolism pathways and the nitrogen metabolism pathway were observed in both intermediate and pathogenic groups (Supplementary dataset S8). This indicated that *Leptospira* species had lost many of the genes encoding carbohydrate metabolism and energy metabolism in the process of evolution into parasitic pathogenic species.

The core genes of nine pathogenic *Leptospira* (1,438) preserved 1,023 genes of MRCA, while gaining a total of 415 new genes (28.8%). After species formation, or in the process of strain diversification, 1,705–2,202 genes were gained versus average 1,382 genes lost, implying diverse host adaptation. The phylogenetic analysis showed that 281 new genes were gained in the MRCA lineage of intermediate and pathogenic groups (Supplementary dataset S9). Although the functions of most newly gained genes were not clear, it was noted that 16 virulence-related genes (such as outer membrane hemin receptor and TCS sensor histidine kinase, penicillin-binding protein, *etc*) were acquired (FDR = 4.4E-5) (Supplementary dataset S9 and S10), hinting that *Leptospira* spp obtained primary virulence at this stage. The three newly-gained two-component sensor histidine kinases separately showed homology to genes of the human pathogen *Legionella pneumophila, Yersinia pestis* and the plant pathogen *Mesorhizobium loti*, implying that lateral gene transfer from other pathogens contributed to the evolution of pathogenic *Leptospira* inside various hosts.

Thereafter, *Leptospira* spp began dramatic genome expansions, with more genes gained than lost. The lineage forming the intermediate group lost 37 MRCA genes but obtained 141 new genes, including six virulence genes (FDR = 0.008). In the pathogenic lineage, before species divergence, 65 MRCA genes were lost and 134 genes were gained. Although most of the gained genes (101) were hypothetical proteins, four of these newly emerged genes were associated with virulence (FDR = 0.02) (Supplementary dataset S10), hinting that species divergence contributed to pathogenic *Leptospira* formation.

Within the pathogenic *Leptospira* lineages, four sub-branches (group I–IV) emerged (Fig. 2B). The gene gain events were more frequent than the gene loss events in the evolutionary process within the four sub-branches. 98 specific genes were gained in the evolution of group I, including three virulence genes (Supplementary dataset S11). Two genes (20046|gene_id_1958 and LA2717) encoded proteins involved in TCS; one protein was predicted to be a receiver component of a two-component response regulator, with 33% identity to a chemotaxis protein of marine bacteria *Pseudoalteromanos ruthenica*; and the other was predicted to be a TCS sensor histidine kinase, with 36% identity to a gene of *Flavobacterium limnosediminis*, an opportunistic pathogen in water. Therefore, the acquired genes involved with TCS components provide further evidence that the TCS of pathogenic species was distinct from the saprophytic group. Such gains were thought to enhance the adaptation abilities of the bacteria in animal host environments. Furthermore, *flhB*, encoding a flagellar biosynthesis protein, was acquired in the lineage. A previous study reported that FlhB shared identity with *Leptospira* FlhB (33.5% identity) and was associated with flagellar expression as well as adhesion to and colonization of the gastric mucosa in *Helicobacter pylori* infections^24^. The acquisition of FlhB may contribute to the ability of this lineage species to infect the host.

For group II, 15 genes including two virulence genes were gained, while 18 genes were lost (Supplementary dataset S11). The gained virulence gene *dam*, encoding DNA adenine methylase, is an essential virulence factor in bacterial pathogens like *Salmonella enterica*, which regulates virulence gene expression^25^ and controls cell envelope integrity^26^. The other virulence gene encoded a protein homologous to β-hemolysin, a surface associated toxin that plays an important role in the pathogenesis of *Staphylococcus aureus* infections^27^. The acquisition of virulence genes and loss of unnecessary genes might contribute to the success of small genome size in high virulence group II *Leptospira*.

The independent lineages represented by *L. alstoni* (group III) gained 504 genes (including three virulence genes) during its evolution, but no gene loss was observed (Supplementary dataset S11). The three *L. alstoni* isolates from *Bombina orientalis* and *Rana nigromaculata*, belonged to *Neobatrachia* species in China. The specific host range and geography implied that *L. alstoni* experienced distinct evolution from the MRCA, and its unique genome features could explain the inability of current multilocus sequence typing schemes to type this species^28^.

Prior to group I, II and III divergence, 51 new genes were gained, including four virulence genes, versus 42 MRCA genes lost. Then, before group II and III divergence, seven new genes were gained and six MRCA genes were lost. *L. kmetyi* (group IV) located beyond group I–III, gained 455 genes (including 14 virulence genes), while losing 102 MRCA genes.

Taken together, gene gains were shown to play crucial roles in shaping the genomic plasticity of pathogenic *Leptospira*. Although most of genes encode unknown products, it assumed that acquisition of genes, especially virulence genes, such as TCS, may contribute to *Leptospira* host adaptation, expanding the range of environments, e.g., allowing for infection of mammals, and permitting the bacteria to spread to novel hosts.

### Horizontal gene transfer and gene duplication facilitated gene gain in pathogenic *Leptospira*

**Figure 3:**
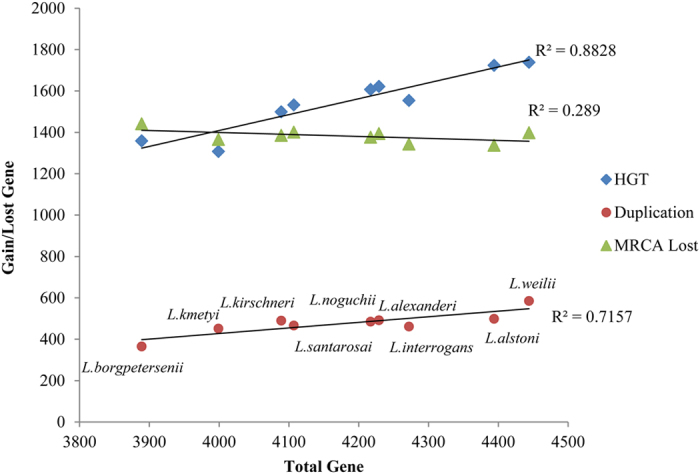
Role of horizontal gene transfer (HGT), gene loss and gene duplication in pathogenic *Leptospira* evolution. Scatter plots of duplication genes, HGT genes and most recent common ancestor (MRCA) lost genes versus the total number of genes in nine pathogenic *Leptospira* species were present. The abscissa represented the gene number of each species, and the ordinate represented the gained or lost gene number.

Bacterial genomes can generate new genes through horizontal gene transfer (HGT) or gene duplication (GD) from other organisms^29^,^30^. We found that both HGT (R^2^ = 0.88) and GD (R^2^ = 0.72) had strong positive correlation with gene number of each species (Fig. 3). 32.7% (*L. kmetyi*) to 39.2% (*L. alstoni*) of the pathogenic *Leptospira* genes were gained through HGT, accounting for most of the new-gained genes. GD contributed 9.4% to 13.2% of *Leptospira* genes (Supplementary dataset S12).

Coinciding with the genome size, *L. borgpetersenii* harbored the lowest number of duplicated genes (365) and HGT genes (1,358) (Supplementary dataset S12). Compared with MRCA, the gene loss in group II species (1,394–1,441) was slightly greater than other species (1,337–1,385), with *L. borgpetersenii* losing the most MRCA genes (1,441) among all the *Leptospira* species. These losses taken together most likely contribute to this specie’s small genome size. Previous studies have shown that adaptation to mammalian hosts by *L. borgpetersenii* is associated with insertion sequence (IS)-mediated genome reduction^11^. Coincidentally, eight copies of IS*1501* and 94 copies of IS*1533* were revealed in *L. borgpetersenii*, while only eight copies of IS*Lin2* and 37 copies of IS*Lin1*, were revealed in *L. interrogans*.

We found that 92 out of the 281 gained genes before the divergence of intermediate and pathogenic *Leptospira* were obtained through HGT, as they exhibited similarities to proteins not found in the *Leptospira* genus. Furthermore, 30 out of 134 pathogenic lineages gained genes that were confirmed as HGT. We also identified a range of genes [51 (1.1%) in *L. interrogans* to 504 (10.6%) in *L. alstoni*] unique to the specific lineage of the pathogenic branch; these genes may have also originated from HGT (Supplementary dataset S13). Subsequently, a significant number of GD occurred after the core-genome was formed in each species, which facilitated adaptation of each species to a specific living environment (Fig. 3 and Supplementary dataset S12).

Although we have demonstrated that gene acquisition contributed substantially to the emergence of pathogenic *Leptospira* from MRCA, it was noted that different species within the pathogenic *Leptospira* lineages underwent distinct evolutionary paths to adapt to host environments. Compared to *L.interrogans*, which is commonly acquired from contaminated surface water, *L. borgpetersenii* is likely to be transmitted by direct contact with contaminated body fluids and fails to survive in nutrient poor environments^11^. As *L. borgpetersenii* and *L. interrogans* can cause lethal infections in non-maintenance hosts^1^, it is believed that the selective gene gain and loss found in different pathogenic species may contribute to the ability of *Leptospira* to retain virulence in diverse conditions.

### Protein family expansion validated gene gain in pathogenic *Leptospira* evolution

To explore gene family evolution among *Leptospira* spp., a comprehensive comparative analysis was first performed for investigating the variations in protein family (PF) numbers of all *Leptospira* species. A total of 2,608 PFs were identified among all the *Leptospira* species, including 952 core families common to all 18 intermediate, saprophytic, and pathogenic species, 1345 dispensable families between any two *Leptospira* species, and 311 unique families found in only one species.

**Figure 4:**
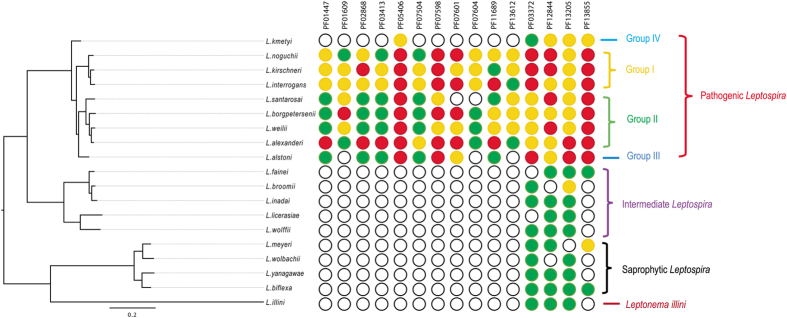
Phylogenetic analysis based on the concatenated orthologous proteins of 18 *Leptospira* species and the outgroup *Leptonema illini* using maximum likelihood method. The complete tree was shown in Supplementary Figure 3. To the right of the tree, average numbers of 14 specific protein families that were not universally shared across all pathogenic species were indicated by different colored circles. White colored: no copies; green colored: 1 copy; orange colored: 2–4 copies; red colored: ≥5 copies. Scale bar indicated an evolutionary distance of 0.2 amino acid substitutions per position.

Further analysis found that 204 PFs existed exclusively in the pathogenic species and were completely absent from all saprophytic and intermediate species (Supplementary dataset S14 and Supplementary Figure 3), suggesting these PFs might be produced from lateral gene transfer. Among them, 12 PFs were present among at least one pathogenic species of each of the four groups while 88 PFs were specific to one pathogenic group (31 in group I, 40 in group II, 0 in group III, and 17 in group IV). Although most PFs paralogs are as yet unknown with respect to their functional role (i.e. they are classified as hypothetical proteins), several PFs associated with virulence were noted. The most representative PF example was the four peptidase families (PF03413, PF07504, PF02868, and PF01447) with remarkably variable paralog numbers. For example, the paralog number of the PF02868 was four in *L. interrogans*, five in *L. kirschneri* and two in *L. noguchii* (a 4-5-2 pattern in group I). And the pattern in group II was five in *L. alexanderi*, one in *L. borgpetersenii*, one in *L. santarosai* and one in *L. weilii* (a 5-1-1-1 pattern in group II), whereas only one protein was observed in *L. alstoni*, and none was observed in *L. kmetyi* (Fig. 4 and Supplementary dataset S14). To successfully multiply and spread in the host, pathogenic leptospires must evolve multiple strategies against host immune defenses, such as the complement system. One paralogous thermolysin protein (LIC13322 of *L. interrogans* serovar Copenhageni str. Fiocruz L1-130) in the PF02868 family, which possessed catalytic domains similar to those of metalloproteases from some pathogens reported to cleave complement proteins, was shown to cleave C3 in human serum, suggesting that LIC13322 could be regarded as an important metalloprotease responsible for inhibition of the complement pathways, as previously observed in pathogenic but not in saprophytic *Leptospira*^31^. However, interestingly, LIC13322 was not able to effectively cleave C2, C4, or factor B, suggesting that other leptospiral thermolysins may be synergistically involved in inactivation of host immune effectors^31^. This might be achieved by expansion of these metalloprotease-associated PFs, like the three paralogs with over 50% amino acids similarity to LIC13322 in the PF02868 family, further enhancing the infectivity of the pathogenic strain. Moreover, among the 204 unique families of pathogenic species, a total of 15 peptidase families were found, suggesting the importance of these PFs in the pathogenesis of *Leptospira*.

In line with previous observations^12^, the expansion events in the PF07598 family among pathogenic species were also identified, and the variable paralog numbers of PF07598 were 20, four and five (on average) in group I, II and III, respectively. No variable paralogs were identified in *L. kmetyi*. There was a trend of gradually increasing expansion from group IV to group I (Fig. 4). Lehmann *et al*.^12^ demonstrated that tissue-specific upregulation of 11 paralogous members in the PF07598 family in the blood and liver, with LA_3490 and LA_3388 upregulated more than 1000-fold *in vivo* in hamsters acutely infected with virulent *L. interrogans* strain Lai.

Furthermore, it is found that the WGR family (PF05406) showed highly variable members, with the highest number in group II (an average of nine in *L. weilii* and ten in *L. santarosai*), six–seven paralogs in group I (six in *L. interrogans*, seven in *L. kirschneri*, and six in *L. noguchii*, a 6-7-6 pattern), eight paralogs in *L. alstoni*, and only two paralogs in *L. kmetyi* (Fig. 4 and Supplementary dataset S14). A previous study reported that LA_0984, a paralog of the WGR family, was significantly highly expressed at multiple different temperature conditions: from 20 °C and 30 °C (environmental temperatures) to 37 °C (simulated human physiological temperatures) and during overnight shift of the temperature from 30 °C to 37 °C or long-term incubation at 30 °C and 37 °C^32^. Thus, these data indicated that LA0984 may be involved in adhesion and entry into the host during the early stages of leptospirosis.

In addition to PFs exhibiting more than one paralog, most of the 204 unique PFs exhibited an expansion pattern of 0–1 when compared to the saprophytic and intermediate species. Some of these families are specific to a single pathogenic species, but most are present in all. However, limited studies have been performed to investigate the paralogs belonging to these PFs in *Leptospira.* We believe that the redundancies of these expanded PFs, exclusively found in pathogenic *Leptospira* species have evolutionary significance and that these PFs likely contribute to *Leptospira*-host interactions as well as subsequent *in vivo* survival and adaptation.

### Massive expansion of virulence-associated protein families

Despite horizontal transferred domains, which usually showed lineage specific expansion, we also revealed expansion of 139 MRCA inherited PFs (Supplementary dataset S15), and most of them showed constant expansion in each pathogenic *Leptospira* species. These observations further highlighted those expansion events in gene families contributed to the evolution of pathogenic *Leptospira*. Among them, the largest gene family was LRR_8, Leucine rich repeat (PF13855), which increased from one member in MRCA to eight in *L. borgpetersenii* and 21 in *L. interrogans* (Fig. 4). LRR_8 family expansion was the result of gene duplication, as we detected four tandem duplication events in LRR_8 gene family of *L. interrogans*, with the largest tandem array consisting of five members. The presence of tandem arrays of gene copies has been taken as the hallmark of gene duplication^33^. The leucine-rich repeat domain containing protein may interact with host cells and contribute to pathogen virulence by stimulating the host inflammatory response^34^. Meanwhile, PF13855 was not expanded in intermediate *Leptospira* (0–2 members), so expansion of this family might play an important role in pathogenic *Leptospira* evolution.

Such gene duplication-associated large scale domain expansions were also observed in other virulence-associated domains (Fig. 4), such as, exo_endo_phos, endonuclease/exonuclease/phosphatase family (PF03372)^35^, HTH_19, helix-turn-helix domain (PF12844)^36^, and bacterial Ig-like domain (PF13205)^37^,^38^,^39^.

Collectively, such redundancy in the expansion of these PFs, whether from HGT or duplication, especially for those associated with virulence, strongly suggested that PF expansion played an important role in the evolution of *Leptospira* pathogenesis. Therefore, it is assumed that this may represent an evolutionary roadmap of virulence in *Leptospira* species.

### Positive selection was associated with host adaption

Although HGT and GD played important roles in forming pathogenic *Leptospira*, half of the genes were inherited from MRCA, suggesting that they may undergo stringent selection pressure in the evolution. To investigate the selective forces acting on the pathogenic genome, we first analyzed the complete spectrum of genetic mutations by comparison of four pairs of *L. interrogans* genomes available that underwent parallel laboratory and host adaptive evolution.

We found a total of 1,712 SNPs across four pairs of differentially evolved strains (Supplementary dataset S16). Generally, parallel adaptive evolution of the four *L. interrogans* strains led to acquisition of mutations in different genes. Interestingly, 28 genes had more than 10 SNPs, although most genes harbor only one SNP (Supplementary Figure 4). These SNPs were mostly found within the coding region and included 308 synonymous mutation SNPs and 932 nonsynonymous mutation SNPs, whereas 472 SNPs were found in the intergenic region (27.57%) (Supplementary dataset S16 and Supplementary Figure 4). Total 161 genes affected by these mutations encoded proteins with a broad range of functions. Of particular interest was LA_3778, a LigB-like protein, which acts as a surface component in bacteria. A total of 131 SNPs (including 64 synonymous mutations and 67 nonsynonymous mutations) were found in this gene. Previous studies reported that LigB may recognize adhesive matrix molecules that allow pathogenic *Leptospira* to bind to host extracellular matrix components, suggesting that it plays a major role in bacterial infection^40^,^41^. Such an overabundance of SNPs on surface protein genes provided evidence for adaptive selection acting on *Leptospira* genomes.

Furthermore, we identified 72 out of the 161 genes under positive selection (Ka/Ks > 1) (Supplementary dataset S17) in the evolutionary process. These positively selected genes were enriched in COG class of defense mechanism and signal transduction mechanism (FDR < 0.05) and pathway of nucleotide metabolism and cell motility (FDR < 0.05) (Supplementary dataset S18). The majority of these genes encoded proteins with functions in cell motility and secretion (10 genes), lipid metabolism (seven genes), and nucleotide transport and metabolism (six genes). The most frequently mutated gene was *spoT*, which mutated in three isolates. SpoT possessed high alarmone guanosine 5′-diphosphate 3′-diphosphate (ppGpp) synthesis activity and played an important role in maintaining the basal level of ppGpp in the cell, which may contribute to regulation of the bacterial stress response^42^ and microbial physiology, like survival, persistence, and virulence by allowing bacteria to adapt to changes in nutrient availability^43^. So mutations in the *spoT* gene may reflect adaptations in the evolutionary strategy from host selective forces.

**Figure 5:**
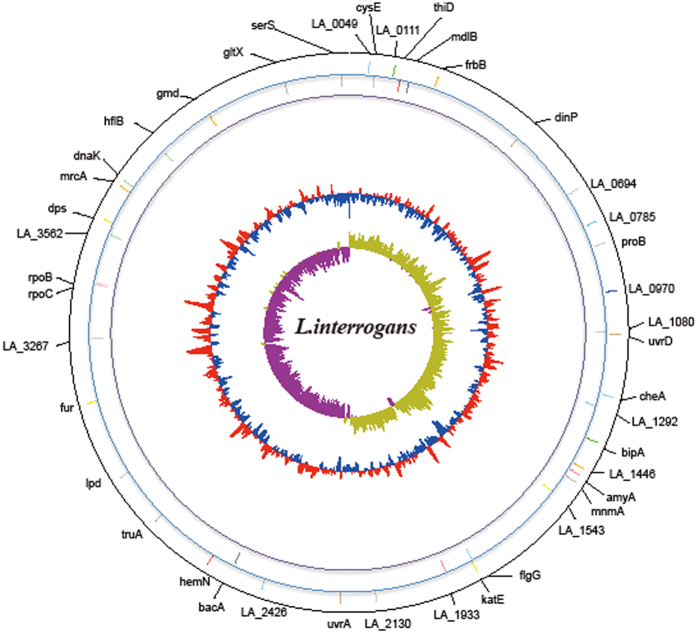
Positively selected virulence genes among the nine pathogenic *Leptospira* species. Moving toward the inside, the first and second circles separately represented the 42 positively selected genes in the plus and minus strands of chromosome I of *L. interrogans* serovar Lai str.56601, and the gene names or loci were labeled outside of the circle. The 3^rd^ and 4^th^ circles represented GC content and GC skew, respectively. Precise molecular details for all mutations are shown in Supplementary dataset S16, S17 and S19.

To further verify the hypothesis that host selection shaped the adaptive evolution of pathogenic *Leptospira*, we also assessed the positive selection of core genes among the nine pathogenic species. A total of 47 virulence genes under positive selection were identified (Fig. 5 and Supplementary dataset S19). In addition, four out of the 47 genes (*LA_1378*-*bipA, LA_1859*-*katE, LA_3419*-*rpoC* and *LB_170*) were under positive selection in laboratory culture (Supplementary dataset S17), indicating an important role of the four genes in pathogenic formation and host adaption.

Previous studies reported that overexpression of GTP-binding protein BipA might disrupt the normal functions of ribosome and impair protein translation and ribosomal biogenesis^13^,^44^. Mutation of *bipA* was postulated to cause the lower level of BipA in the virulent strain 56601, and ultimately lead to more active protein translation in the highly virulent strain^13^. So positive selection of *bipA* in pathogenic species might accelerate its mutation and help the pathogenic *Leptospira* survive the host environment. KatE is the only annotated catalase found within pathogenic *Leptospira* species and is required for resistance to oxidative killing, which is usually induced by host innate immune response^45^. Pathogenic *L. interrogans* bacteria had a 50-fold-higher survival rate than saprophytic *L. biflexa* bacteria under H_2_O_2_-induced oxidative stress due to the expression of *katE*^45^. The positive selection of *katE* emphasized its important role in helping pathogenic *Leptospira* species disarm host defense mechanisms. *LB_170* encoded capsular polysaccharide biosynthesis protein. Genes related to the biosynthesis of cell wall capsular polysaccharides were postulated to have a potential role in pathogenesis and virulence^9^. Cell surface capsular polysaccharides may also be important in the survival of pathogenic *leptospira* in the environment outside the host, protecting them from hydric and osmotic stresses. The positive selection of *LB_170* may increase the virulence of pathogenic *leptospira*. Taken together, the impacts of different pathogenic species from positive selection were different, suggesting that each species may have undergone distinct evolutionary pressures from different host.

## Conclusion

This comparative genomics study revealed that the diverse global populations of pathogenic *Leptospira* species had large and diverse genomes. The open pan-genome also reflected the high genomic variability among species. Such high genomic diversity can be attributed to massive gene gain and loss events, implicating that each species had to cope with specific niche conditions with changing host environments. More genes were lost than gained before separation of pathogenic and intermediate groups, while more genes were gained than lost in the separate evolution of each pathogenic *Leptospira* species. The comparative phylogenomics analysis provided evidence that horizontal gene transfer and gene duplication events facilitated the stepwise acquisition of virulence factors, such as distinct TCS, in the emergence of pathogenic *Leptospira* from MRCA. More importantly, special virulence-related PF expansions such as metalloproteases-associated paralogs, played an important role in the evolution of *Leptospira* pathogenesis. Therefore, this may be an evolutionary mechanism mediating the virulence of *Leptospira* species. Based on genomic comparisons of four pairs of strains that underwent parallel laboratory and host adaptive evolution, we found that most genes (64.28%, 72/112) affected by mutations were under positive selection. Positive selection test of core genes among nine pathogenic species also revealed 47 virulence genes under positive selection, including *bipA, katE*, etc., which provided more evidenceto support that positive selective pressure played an important role in adaptation of bacteria to hosts. To our knowledge, this is first comprehensive genomic study that included pathogenic *Leptospira* isolates from worldwide sources. These results will improve our understanding of the global diversity and virulence evolution of this genus of spirochetes, and pave the way for developing strategies aimed at controlling this neglected tropical disease.

## Methods

### Isolates

The strains used in this study are listed in Supplementary dataset S1. *Leptospira* were grown to late log phase and harvested by centrifugation. Genomic DNA was extracted using a Wizard Genomic DNA Purification Kit (Promega, Southampton, UK) following the manufacturer’s instructions. Furthermore, a *Leptonema illini* isolate was included for comparison and analysis.

### Genome sequencing and assembly

The large-scale str.J50 and str.20046 genomes were sequenced using a GS FLX 454 system with an 800 bp pair-end library following standard protocols^46^. The total of 275,814 reads in str.20046 (average length of 305 bp) and 307,424 reads in str.J50 (average length of 310 bp) were produced, respectively. A 300-bp paired-end library was separately constructed for each purified DNA sample from other strains following the standard Illumina paired-end protocol. Cluster generation was performed in C-bot, and sequencing was performed on the Illumina Hiseq 2500 with 150 cycles. On average, 1.64 Gb of Illumina reads were produced for each genome. Genome assembly was performed using the program Velvet 1.2.03^47^ with the following custom parameters: hash-length = 81–111 and coverage cut-off = 30. The genome assemblies were aligned in a pair-wise fashion using Mauve^48^ and using *L. interrogan*s serovar Lai 56601 as reference.

### Genome annotation and analysis

Putative protein-coding sequences were determined by combining the prediction results in the GeneMark program^49^. Functional annotation of CDS was performed by searching the NCBI nonredundant protein database. COG assignment^50^ were performed by RPS-BLAST using the NCBI CDD library^51^. Protein domain (PF) predication was performed by InterproScan^52^. Insertion sequences (*ISs*) were identified using all *Leptospira ISs* downloaded from IS finder database (http://www-is.biotoul.fr/)^53^. Metabolic pathways were constructed using the KEGG database^54^. The subcellular localization of the proteins was predicted using the PSORTb program (v2.0.1)^55^. Previously published genomes of spirochetes were also included in the phylogenetic analysis (Supplementary dataset S1)^7^,^8^,^9^,^10^,^11^,^12^,^13^. Comparative genomics analysis was performed using Mauve^48^, and a phylogenetic tree was constructed using PHYML^56^ by concatenating orthologs based on protein sequences in each genome.

### Pan-genome analysis and positive selection

Pan-genome analysis was performed using PGAP 1.11^57^ with the MP method using the following settings: intraspecies coverage, 50%; intraspecies identity, 50%; interspecies coverage 50%; and interspecies identity 20%. The intergenomic distances of different species were calculated using GGDC 2.0^58^. To investigate the selective forces acting on the pathogenic genome, four pairs of *L. interrogans* isolates from the same source but passaged under different conditions (artificial culture medium and an animal model) for more than twenty years were also sequencing, and the genomes were compared in parallel. The four pairs of strains were as follows: 56001-V and 56001, 56006-V and 56006, 56008-V and 56008, and 56009-V and 56009 (V presents strains passaged via animal infection). The nonsynonymous (Ka) and synonymous (Ks) substitution rates of orthologous genes between the four pairs of strains were calculated by KaKs_Calculator through model averaging^59^. A likelihood-ratio test for positive selection was conducted with codeml from PAML software version 4.7^60^, with p-value <0.05 (LRTs comparing M1 and M2) as presence of sites evolving by positive selection.

### Virulence gene identification

We used VFDB database^61^ and MvirDB^62^ to identify virulence factors.

### Accession numbers

All the raw data were deposited in SRA under accession number SRP045203, SRP045341, SRP045392-045394, SRP045397-045400. The Whole Genome Shotgun project had been deposited at DDBJ/EMBL/GenBank under the accession JQOL00000000-JQSB00000000. The versions described in this paper were version JQOL01000000-JQSB01000000.

## Additional Information

**How to cite this article**: Xu, Y. *et al*. Whole genome sequencing revealed host adaptation-focused genomic plasticity of pathogenic *Leptospira. Sci. Rep.* **6**, 20020; doi: 10.1038/srep20020 (2016).

## Supplementary Material

Supplementary Information

Supplementary dataset S1

Supplementary dataset S2

Supplementary dataset S3

Supplementary dataset S4

Supplementary dataset S5

Supplementary dataset S6

Supplementary dataset S7

Supplementary dataset S8

Supplementary dataset S9

Supplementary dataset S10

Supplementary dataset S11

Supplementary dataset S12

Supplementary dataset S13

Supplementary dataset S14

Supplementary dataset S15

Supplementary dataset S16

Supplementary dataset S17

Supplementary dataset S18

Supplementary dataset S19

## Figures and Tables

**Table 1:** Genetic features of the *Leptospira* species sequenced in this study.

**Spceies**	**Genome Size (Mb)**	**Gene Number**	**Core Genome**	**Pan Genome**	**GC content**
*L.alexanderi* (n = 5)	4.14 (4.10–4.16)	4605 (4500–4678)	3062	7369	40.25% (40.31%−40.28%)
*L.alstoni* (n = 3)	4.40 (4.29–4.45)	4734 (4574–4819)	3711	5645	42.50% (42.61%−42.54%)
*L.borgpetersenii* (n = 10)	3.88 (3.79–3.96)	4281 (4191–4443)	2979	8987	40.11% (40.01%−40.30%)
*L.interrogans* (n = 62)	4.77 (4.40–5.02)	4554 (4104–4990)	2500	25725	34.90% (35.26%−34.99%)
*L.kirschneri* (n = 2)	4.54 (4.50–4.59)	4376 (4284–4467)	3254	5104	35.85% (36.24%−36.05%)
*L.noguchii* (n = 5)	4.67 (4.59–4.73)	4534 (4413–4770)	3071	7348	35.27% (35.62%−35.45%)
*L.santarosai* (n = 7)	3.99 (3.90–4.12)	4451 (4280–4660)	2985	8174	41.60% (41.85%−41.78%)
*L.weilii* (n = 8)	4.31 (4.06–4.46)	4816 (4590–5004)	2806	9114	40.41% (40.86%−40.69%)
*Leptonema illini* (n = 1)	4.44	4404	4404	4404	54.34%
