# Supplementary material for: Whole genome sequencing revealed host adaptation-focused genomic plasticity of pathogenic Leptospira
Source: Sci Rep. 2016 Feb 2;6:20020. doi: 10.1038/srep20020 (PMC4735792; doi:10.1038/srep20020)
Supplement: Supplementary Information [file srep20020-s1.pdf]

Supplementary Information

Whole genome sequencing revealed host adaptation-focused genomic plasticity of pathogenic *Leptospira*

Yinghua Xu[1#], Yongzhang Zhu[2#],Yuezhu Wang[4#], Yung-Fu Chang[5], Ying Zhang[1], Xiugao Jiang[7], Xuran Zhuang[2], Yongqiang Zhu[4], Jinlong Zhang[1], Lingbing Zeng[2], MinjunYang[4], Shijun Li[8], Shengyue Wang[4], Qiang Ye[1], Xiaofang Xin[1], Guoping Zhao[4,6], Huajun Zheng[3,4*], Xiaokui Guo[2*], Junzhi Wang[1*]

1. Key Laboratory of the Ministry of Health for Research on Quality and Standardization of Biotech Products, National Institutes of Food and Drug Control, Beijing 100050, People's Republic of China

2. Department of Microbiology and Immunology, Institute of Medical Science, Shanghai Jiao Tong University School of Medicine, 280 South Chongqing Road, Shanghai 200025, People's Republic of China

3. Laboratory of Medical Foods, Shanghai Institute of Planned Parenthood Research, 2140 Xie-Tu Road, Shanghai 200032, People's Republic of China

4. Shanghai-MOST Key Laboratory of Health and Disease Genomics, Chinese National Human Genome Center at Shanghai, Shanghai Zhang Jiang Hi-TechPark, 250 Bi-Bo Road, Shanghai 201203, People's Republic of China

5. Department of Population Medicine and Diagnostic Sciences, Cornell University, Ithaca, New York, 14853, United States of America

6. Key Laboratory of Synthetic Biology, Institute of Plant Physiology and Ecology, Shanghai Institutes for Biological Sciences, Chinese Academy of Sciences, Shanghai 200032, People's Republic of China

7. State Key Laboratory for Infectious Disease Prevention and Control, National Institute for Communicable Disease Control and Prevention, Chinese Centre for Disease Control and Prevention, 155 Changbai Road, Changping District, 102206 Beijing, People's Republic of China

8. Guizhou Provincial Centre for Disease Control and Prevention, 73 Bageyan Road, Guiyang 550004, People's Republic of China

***Corresponding author:** Dr. Junzhi Wang, E-mail: wangjz@nifdc.org.cn; Dr. Xiaokui Guo, E-mail: microbiology@sjtu.edu.cn; Dr. Huajun Zheng, E-mail: zhenghj@chgc.sh.cn

#These authors contributed equal to this work

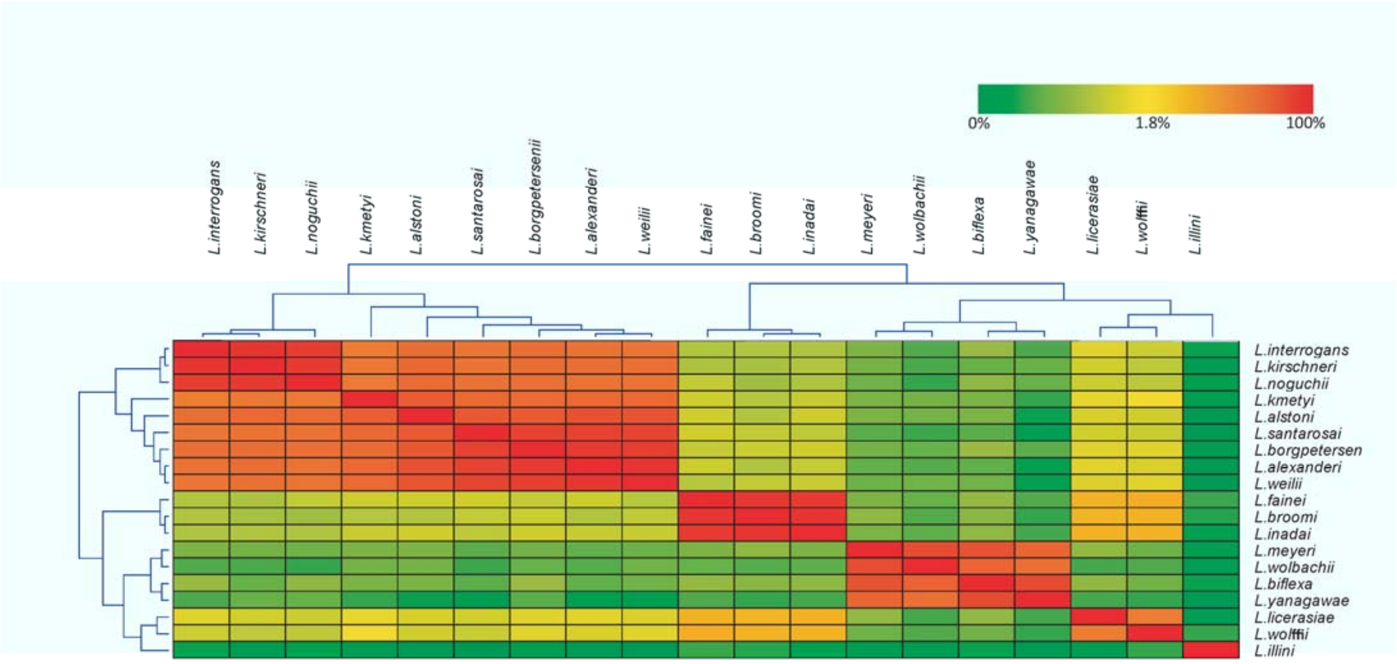

Supplementary Figure 1. Whole-genome similarity heatmap, constructed by Genesis[1]. The similarity between any pair of species was displayed by sum of all identities found in HSPs divided by total genome length using GGDC[2]. At the top of the figure, an indication of the relatedness between genomes is given. Strains are listed in the same order on the x- and y-axes.

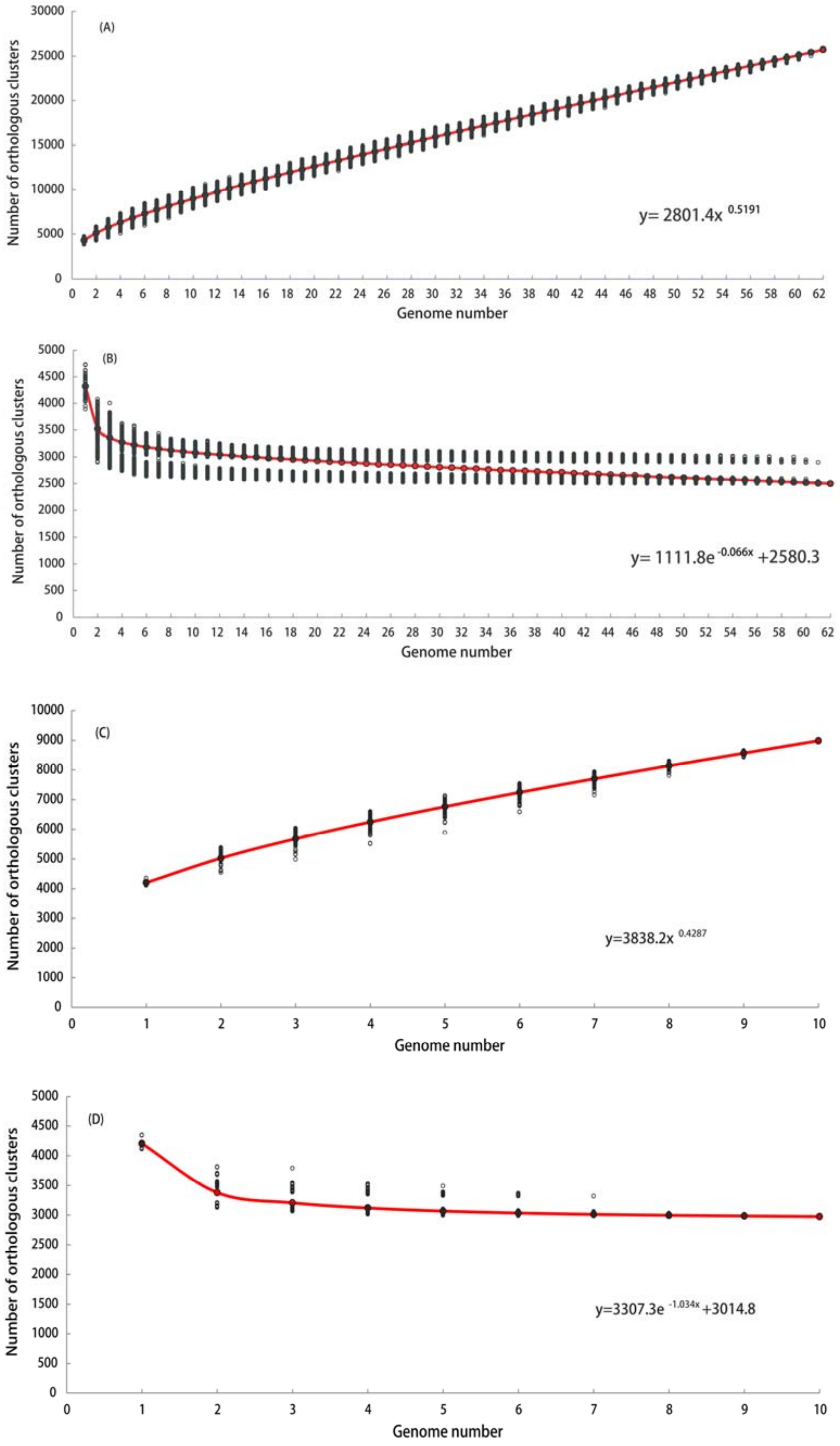

Supplementary Figure 2. Accumulation curves for the pan-genome and core genome of the *L. interrogans* and *L. borgpetersenii*. (A) Pan-genome of *L. interrogans*; (B) Core-genome of *L. interrogans*; (C) Pan-genome of *L. borgpetersenii*; (D) Core-genome of *L. borgpetersenii*. Circles denote values obtained for different strain combinations. The red curve of panel A and C was a least squares fit of the power law $y = k x^{\gamma}$ to medians, and the red curve of panel B and D was least squares fit of the exponential decay $y = kc \exp[-x/tc] + \Omega$ to medians, with Ω representing extrapolated core genome size.

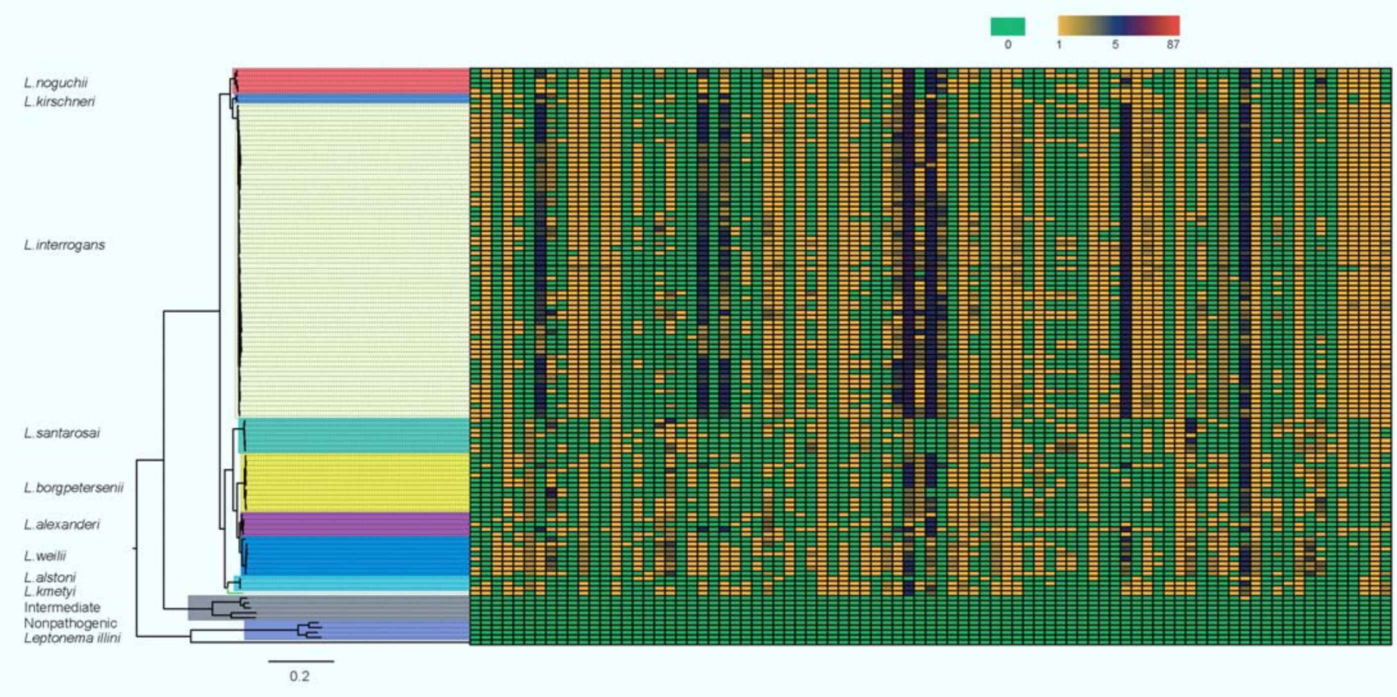

Supplementary Figure 3. Phylogenetic analysis based on the concatenated alignment of orthologous proteins of all *Leptospira* isolates. The tree was rooted with *Leptonema illini* as the outgroup. All species were clearly separated in the orthologous tree. To the right of the tree, the copy numbers of 85 specific protein families from each strain were shown using heatmap. Scale bar indicates an evolutionary distance of 0.2 amino acid substitutions per position.

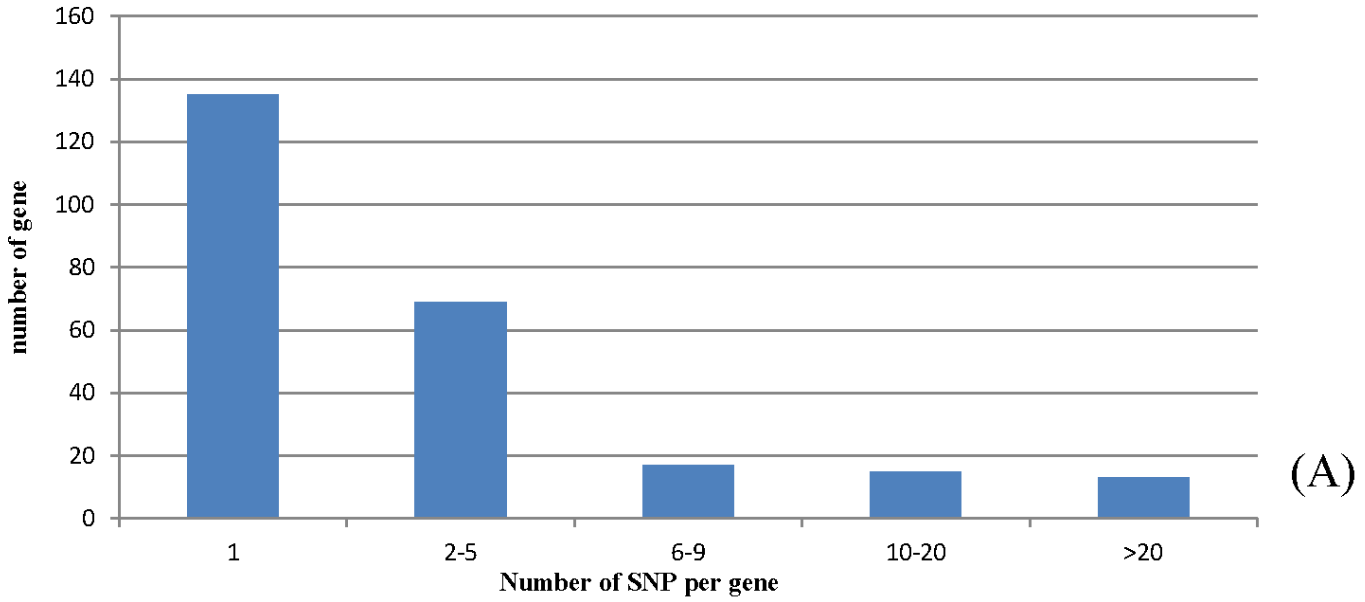

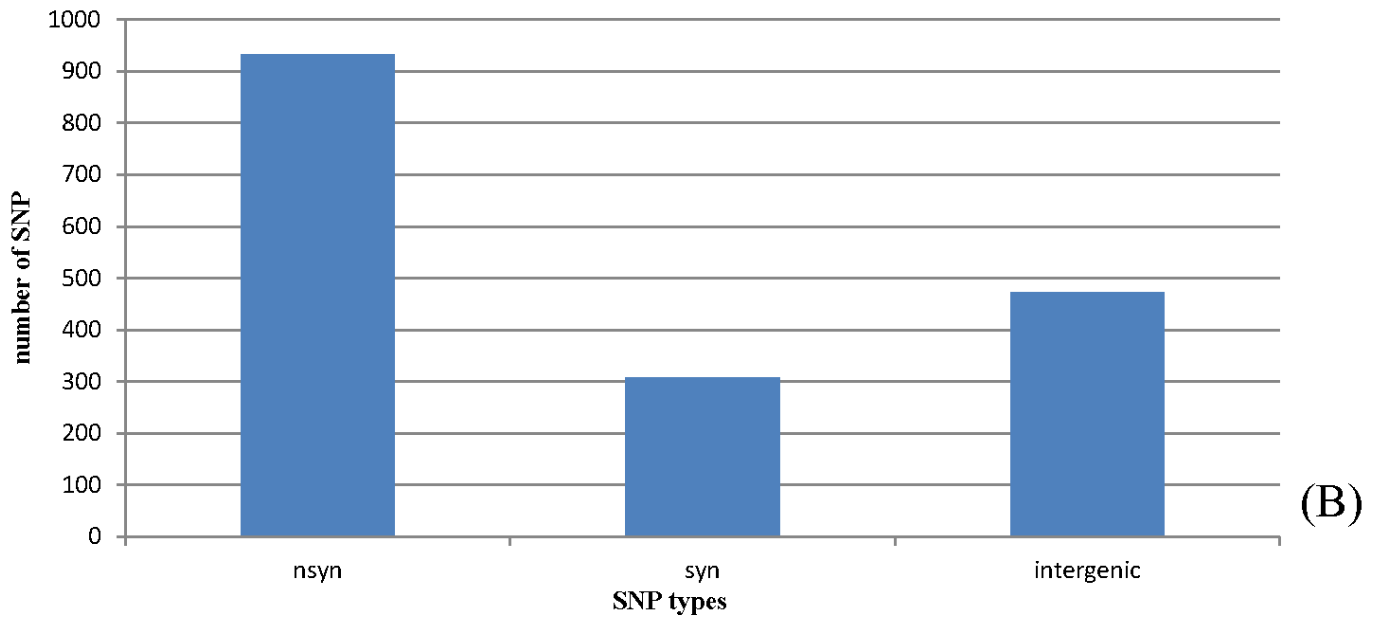

Supplementary Figure 4. Distribution of the number of SNPs per gene (A) and per SNP type (B). Note: syn: synonymous mutation; nsyn: nonsynonymous mutation; intergenic: mutation in an intergenic region.

Reference

1. Sturn, A., Quackenbush, J. & Trajanoski, Z. Genesis: cluster analysis of microarray data. *Bioinformatics* **18**, 207-208 (2002).
2. Meier-Kolthoff, J.P., Auch, A.F., Klenk, H.P. & Goker, M. Genome sequence-based species delimitation with confidence intervals and improved distance functions. *BMC Bioinformatics* **14**, 60 (2013).
